# High throughput procedure utilising chlorophyll fluorescence imaging to phenotype dynamic photosynthesis and photoprotection in leaves under controlled gaseous conditions

**DOI:** 10.1186/s13007-019-0485-x

**Published:** 2019-09-18

**Authors:** Lorna McAusland, Jonathan A. Atkinson, Tracy Lawson, Erik H. Murchie

**Affiliations:** 1 Division of Plant and Crop Science, School of Biosciences, University of Nottingham, Sutton Bonington Campus, Sutton Bonington, Leicestershire LE12 5RD UK; 2 School of Life Sciences, University of Essex, Wivenhoe Park, Colchester, Essex CO4 3SQ UK

**Keywords:** Photosynthesis, Photo-protection, Chlorophyll fluorescence, Dynamic, Phenotyping, Imaging, Wheat

## Abstract

**Background:**

As yields of major crops such as wheat (*T. aestivum*) have begun to plateau in recent years, there is growing pressure to efficiently phenotype large populations for traits associated with genetic advancement in yield. Photosynthesis encompasses a range of steady state and dynamic traits that are key targets for raising Radiation Use Efficiency (RUE), biomass production and grain yield in crops. Traditional methodologies to assess the full range of responses of photosynthesis, such a leaf gas exchange, are slow and limited to one leaf (or part of a leaf) per instrument. Due to constraints imposed by time, equipment and plant size, photosynthetic data is often collected at one or two phenological stages and in response to limited environmental conditions.

**Results:**

Here we describe a high throughput procedure utilising chlorophyll fluorescence imaging to phenotype dynamic photosynthesis and photoprotection in excised leaves under controlled gaseous conditions. When measured throughout the day, no significant differences (*P* > 0.081) were observed between the responses of excised and intact leaves. Using excised leaves, the response of three cultivars of *T. aestivum* to a user—defined dynamic lighting regime was examined. Cultivar specific differences were observed for maximum PSII efficiency (*F*_v_′/*F*_m_′—*P* < 0.01) and PSII operating efficiency (*F*_q_′/*F*_m_′—*P* = 0.04) under both low and high light. In addition, the rate of induction and relaxation of non-photochemical quenching (NPQ) was also cultivar specific. A specialised imaging chamber was designed and built in-house to maintain gaseous conditions around excised leaf sections. The purpose of this is to manipulate electron sinks such as photorespiration. The stability of carbon dioxide (CO_2_) and oxygen (O_2_) was monitored inside the chambers and found to be within ± 4.5% and ± 1% of the mean respectively. To test the chamber, *T. aestivum* ‘Pavon76’ leaf sections were measured under at 20 and 200 mmol mol^−1^ O_2_ and ambient [CO_2_] during a light response curve. The *F*_v_′/*F*_m_′was significantly higher (*P* < 0.05) under low [O_2_] for the majority of light intensities while values of NPQ and the proportion of open PSII reaction centers (qP) were significantly lower under > 130 μmol m^−2^ s^−1^ photosynthetic photon flux density (PPFD).

**Conclusions:**

Here we demonstrate the development of a high-throughput (> 500 samples day^−1^) method for phenotyping photosynthetic and photo-protective parameters in a dynamic light environment. The technique exploits chlorophyll fluorescence imaging in a specifically designed chamber, enabling controlled gaseous environment around leaf sections. In addition, we have demonstrated that leaf sections do not different from intact plant material even > 3 h after sampling, thus enabling transportation of material of interest from the field to this laboratory based platform. The methodologies described here allow rapid, custom screening of field material for variation in photosynthetic processes.

## Background

Plant phenotyping or phenomics is defined as the high throughput quantification of relevant plant and crop traits for research or breeding purposes and includes growth, morphology, photosynthesis and biochemistry, often exploiting the most recent advances in image analysis [1]. While the area of genomic profiling and DNA sequencing have advanced rapidly in recent years, phenotyping methods are still entering into common research practice to complement large-scale crop improvement and still developing in terms of throughput and resolution [2–5]. Consequently, phenotyping is still considered a bottle-neck for advancing biomass and yield in crop species where such techniques can used to identify novel breeding traits but currently take considerable time to collect and analyse [2, 6, 7]. With the added complexity of environmental plasticity and plethora of phenotypic parameters to choose from, plant phenomics benefits from large interdisciplinary collaborations where methodologies can be rapidly disseminated. The ultimate aim is the integrated assessment of key phenotypic traits in combination with application of genomic data of large plant populations—a prerequisite for linkage mapping or genome-wide association mapping of quantitative trait loci; correlating sections of DNA with quantitative phenomic attributes [8–12].

Improving photosynthesis in major crop species is a key phenotypic trait associated with higher biomass production, grain yield and Radiation Use Efficiency (RUE—biomass produced per unit radiation intercepted). Currently, it is estimated that C3 crops convert between 1 and 2% of solar energy to biomass of a possible 4.5%. CO_2_ enrichment studies (FACE) [13, 14] and Zhu et al. [15] have demonstrated that modern crop species have the capacity to improve photosynthetic efficiency by 2–3.5% [16]. However, photosynthesis is not a single trait, but rather the product of a series of linked processes including biochemical capacity (e.g. carboxylation and electron transport), anatomical characteristics (e.g. stomatal and mesophyll conductance) and the ability of the plant to interact with dynamic environmental conditions [17]. Each of these processes can be assessed using techniques such as gas exchange, spectroscopy and microscopy but these tend to be low throughput, slow and labour intensive and often only assessed at a single time point. Phenotyping cereal crops presents additional problems including the size of the plant, its 3-dimensional complexity and the need to capture the correct developmental stage [18, 19]. For example, the size of a wheat plant and the complex array of leaves in space with multiple occlusions creates problems for applying chlorophyll fluorescence imaging technology where leaves should be illuminated evenly, the distance of plant to sensor needs to be known with some precision and a period of dark adaptation of leaves may be required. With high numbers of lines required for genetic mapping or crossing programs, traditional physiological techniques often fall short of providing the large volumes of data required for accurate predictions required to improve molecular breeding strategies.

Chlorophyll fluorescence (CF) is a rapid and non-invasive, high resolution technique to determine changes in photochemistry through monitoring the fluorescence emission of photosystem II (PSII) in situ [20–24]. For example, at 2% oxygen, photorespiration is virtually eliminated; therefore, the products of linear electron transport can be directly related to those used in CO_2_ assimilation. Under these conditions, the PSII operating efficiency (*F*_q_′/*F*_m_′) is positively and directly correlated with the rate of CO_2_ assimilation [23, 25, 26]. Measurements of chlorophyll fluorescence are non-invasive and can be used in imaging setups [21, 27], in combination with other techniques [28] and even remotely on a large scale [29]. Devices can be cheap and simple [30], or highly complex and linked to gas exchange to measure processes such as photorespiration under low oxygen [20].

Imaging leaves or whole plants using chlorophyll fluorescence provides data on the spatial and temporal response of PSII to fluctuating environmental conditions such as Photosynthetic Photon Flux Density (PPFD), CO_2_ and O_2_. In the field, plants are continuously responding to changing environmental conditions and it is increasingly apparent that assessment of *dynamic* responses is key to the identification of lines that either maintain or improve yields in the field [17]. Recent studies have emphatically shown the importance of the dynamic photosynthetic responses to crop yield and plant productivity both with gas exchange measurements [31, 32] and chlorophyll fluorescence [33], leading to identification of lines of interest. For example, chlorophyll fluorescence has been used to: determine carbon assimilation rate under low [O_2_] [27]; to analyse transgenic lines for improved photo-protective de-activation kinetics and improved crop biomass [33] and finally, to assessing plants in which the photorespiratory pathway has been manipulated leading to improved yield [34, 35].

Here, we describe a novel method for screening photosynthetic efficiency in wheat leaves and show for the first time the feasibility of using excised leaf tissue as a highly convenient means to achieve accurate, rapid quantification of complex dynamic shifts in photosynthetic efficiency and photoprotection in a high throughput fashion. Importantly this overcomes some of the practical limitations of phenotyping large numbers of field grown cereal plants. We present designs for a custom-adapted imaging chamber to applying user-defined gas concentrations within the measurement system. The methodology described here facilitates the screening of 100+ individual mature plants simultaneously (several hundred plants day^−1^) in response to complex, custom dynamic light protocols for temporal assessment of PSII processes.

## Methods

### Overview of the screening pipeline

**Fig. 1:**
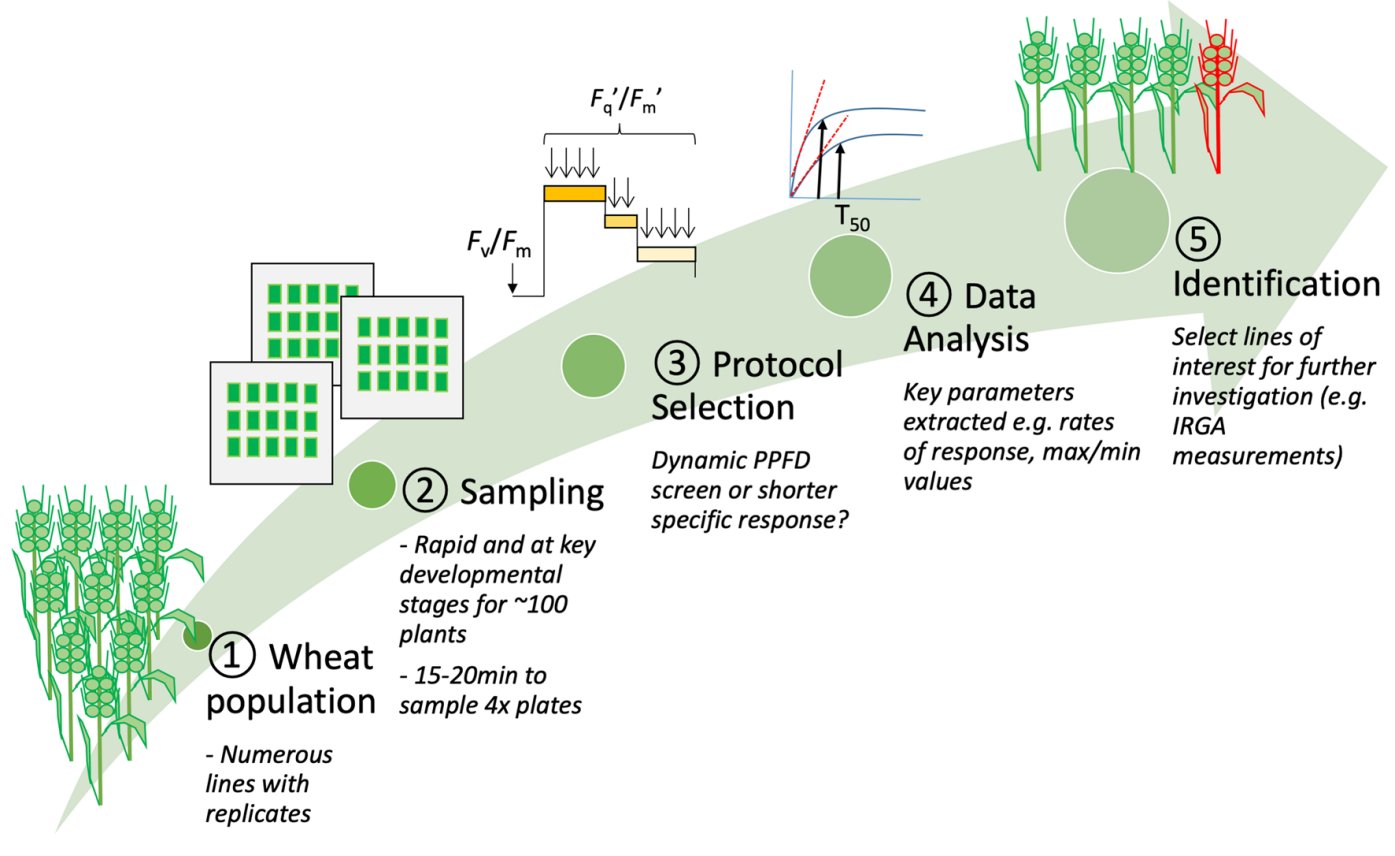
A diagrammatic representation of the proposed screening pipeline, from selecting a large wheat population of interest, rapidly sampling leaf material, to running a user defined protocol and ultimately selecting lines of interest based on chlorophyll fluorescence parameters. These lines could then be investigated for mechanistic differences in carbon acquisition or introduced straight into pre-breeding and breeding programs

An overview of the screening pipeline using a large population of wheat plants is shown in Fig. 1. At a set developmental stage (e.g. flag leaf emergence—growth stage 41–42 according to the Decimal Code System [36]), approximately 2 cm^2^ leaf material is excised and placed on damp paper towel. For transportation, these samples are positioned between two sheets of glass and placed in an insulated box to prevent large temperature changes. Sampling of 100 plants takes between 15 and 20 min. The plates are then transported to a customised FluorCam CF imager (FC800-222, Photon systems instruments, Drasov, Czech Republic) and arranged inside the custom chamber (see ‘Imaging Chamber Design’ for details of customisation). During a standard screen the leaf sections are dark adapted for 1 h. This rapid sampling procedure allows numerous leaf sections from glasshouse or field grown plants to be imaged simultaneously. The PPFD protocol can either be manufacturer or user defined. Using the custom imaging chambers (see ‘Imaging Chamber Design’), leaf sections can also be screened under controlled gaseous conditions (See ‘Timing and Control of Gases’). Typically, the gases are turned on 45 min into the hour and the pressure and concentrations of CO_2_ and O_2_ are monitored using sensors (Qubit Systems Inc., Kingston, Canada). On the hour (and after conditions are constant within the chamber) the selected protocol is run.

### Technical properties of the chlorophyll fluorescence imager

Chlorophyll fluorescence imaging was performed using a customised FluorCam imaging fluorometer fitted with a white and red LED panel (Additional file 1: Fig. S1). Shutter time and sensitivity of the charge-coupled device (CCD) were adjusted in accordance with sample. The FluorCam is located in a temperature controlled dark room maintained between 20 and 22 °C.

Modern fluorometers commonly use a modulated light source at a known frequency to induce chlorophyll fluorescence—otherwise known as pulse-amplitude modulated (PAM) fluorescence—where the detector is set to measure at the same frequency as the excitation [37]. This methodology allows measurements to occur when the plant is illuminated. During a typical measurement, the plant is dark adapted (between 20 and 60 min) to allow maximal plastoquinone A (Q_A_) oxidation after which the leaf is exposed to a saturating flash of light that maximally reduces Q_A_, closing all PSII reaction centres. This procedure gives a maximum fluorescence value (*F*_m_) and, in the light, allows the separation of the photochemical (e.g. PSII operating efficiency—*F*_q_′/*F*_m_′) and non-photochemical (e.g. Non-photochemical quenching—NPQ) processes in the leaf under specific photosynthetic photon flux density (PPFD) conditions. The parameter *F*_q_′/*F*_m_′, also termed ɸPSII or quantum yield (QY), is a measure of the proportion of absorbed light utilised by PSII and therefore can also be used, in combination with measurements of leaf absorbance, to calculate linear electron transport rate (ETR). These parameters (Table 1) are key in the identification of differences between different lines, treatments (biotic or abiotic) or genotypes [20–22, 38, 39]. Many instruments are available for assessment of these parameters either as spot measurements or as images. The benefit of imaging chlorophyll fluorescence is the ability to analyse both temporal and spatial variation in PSII efficiency [28].

**Table 1:** Commonly used abbreviations and equations employed when measuring chlorophyll fluorescence

Parameter	Formula	Definition
*F*, *F*′, *F*_s_′	n/a	Steady state fluorescence emission from dark- or light-adapted (‘) leaf, respectively. *F*′ is sometimes referred to as *F*_s_′ when at steady state.
*F*_*m*_*, F*_*m*_′	n/a	Maximal chlorophyll fluorescence measured in a dark- or light-adapted state respectively
*F*_*o*_ *, F*_*o*_ *′*	n/a	Minimal chlorophyll fluorescence measured in a dark- or light-adapted state respectively
*F*_*v*_ *, F*_*v*_ *′*	n/a	Variable chlorophyll fluorescence measured as the difference between dark- or light-adapted *F*_m_/*F*_m_′ and *F*_o_/*F*_o_′.
*F*_v_/*F*_m_	(*F*_m_–*F*_o_)/*F*_m_	Maximum quantum efficiency of PSII.
*F*_v_′/*F*_m_′	(*F*_m_′–*F*_o_′)/*F*_m_′	Maximum efficiency of PSII in the light.
*F*_q_′/*F*_m_′	(*F*_m_′–*F*′)/*F*_m_′	PSII operating efficiency: the quantum efficiency of PSII electron transport in the light. AKA ΦPSII, quantum yield or Δ*F*/*F*_m_′
ETR or *J*	ΦPSII (AKA *F*_q_′/*F*_m_′) × PPFDa × (0.5)	Linear electron transport rate; where PPFDa is absorbed light (μmol m^−2^ s^−1^) and 0.5 is a factor that accounts for the partitioning of energy between PSII and PSI.
NPQ	(*F*_m_–*F*_m_′)/*F*_m_′	Non-photochemical quenching: estimates the rate constant for heat loss from PSII.
qL	(*F*_q_′/*F*_v_′)/(*F*_o_′/*F*′)	Estimates the fraction of open PSII centers (Q_A_ oxidized); considered a more accurate indicator of the PSII redox state than qP
qP	(*F*_m_′–*F*′)/(*F*_m_′–*F*_o_′) AKA *F*_q_′/*F*_v_′	Photochemical quenching: relates PSII maximum efficiency to operating efficiency. Non-linearly related to proportion of PSII centers that are open. 1–qP has also been used to denote proportion of closed centers

A summary table of the commonly used chlorophyll fluorescence parameters and corresponding equations. For a more comprehensive review please refer to Murchie and Lawson [21], Baker [20] and Maxwell and Johnson [22]

### Custom imaging chamber for controlling measuring environment

**Fig. 2:**
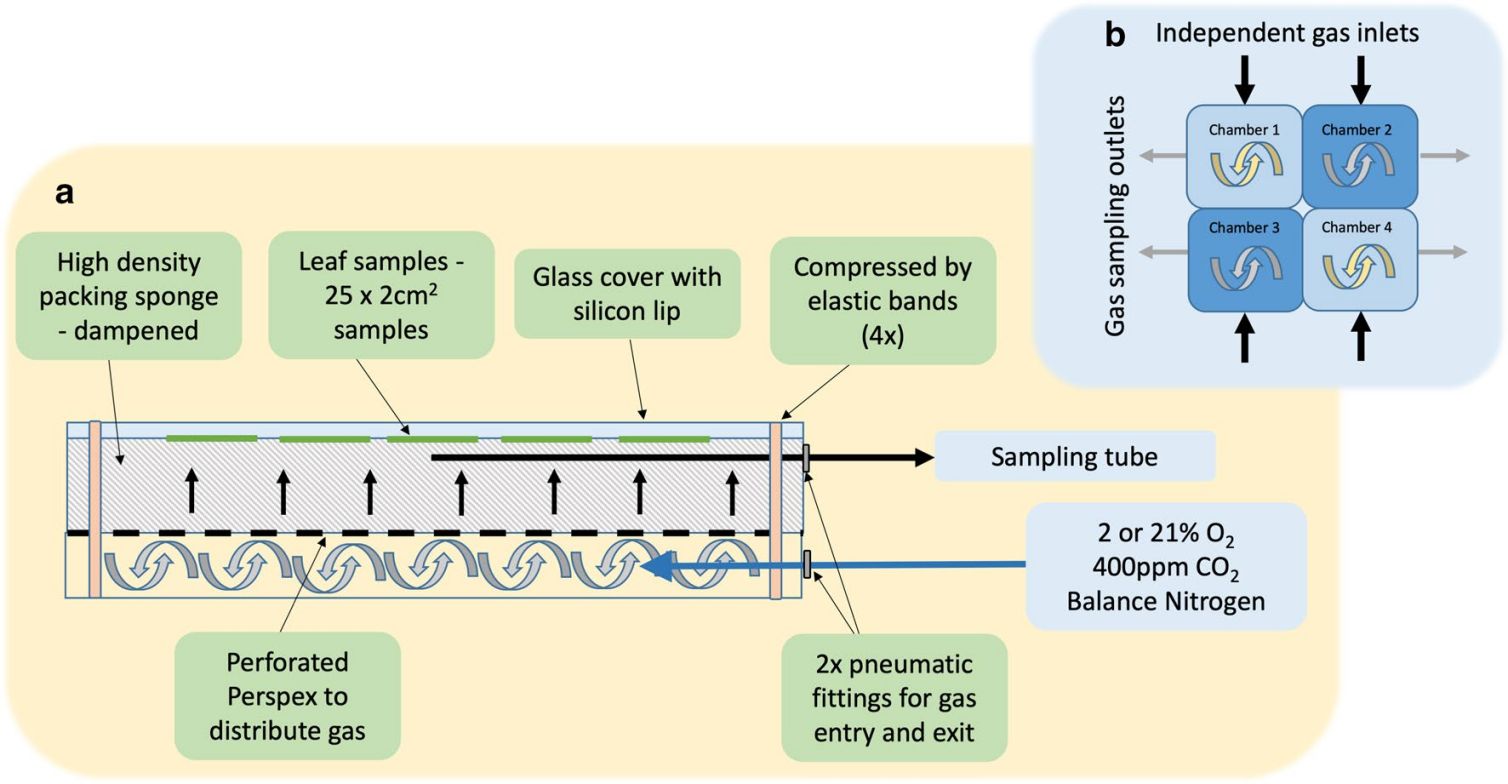
A schematic of the imaging chamber for use with the chlorophyll fluorescence imaging setup. Sections of leaf material are placed atop dampened packing sponge using a thin glass plate affixed using rubber bands (**a**). Gases are fed into a chamber below the sponge and forced through a perforated Perspex separator for even distribution. A tube located in the upper section samples the gases at leaf level to determine the concentrations of oxygen and carbon dioxide. The chamber is designed to sit as one of four chambers under the camera (**b**) allowing the user more flexibility with measurements; either treating each chamber as a separate experiment with separate gas concentrations for screening the response of ~ 25–30 leaf sections or for screening the response of 100–150 sections under singular gas concentrations in one measuring session

**Fig. 3:**
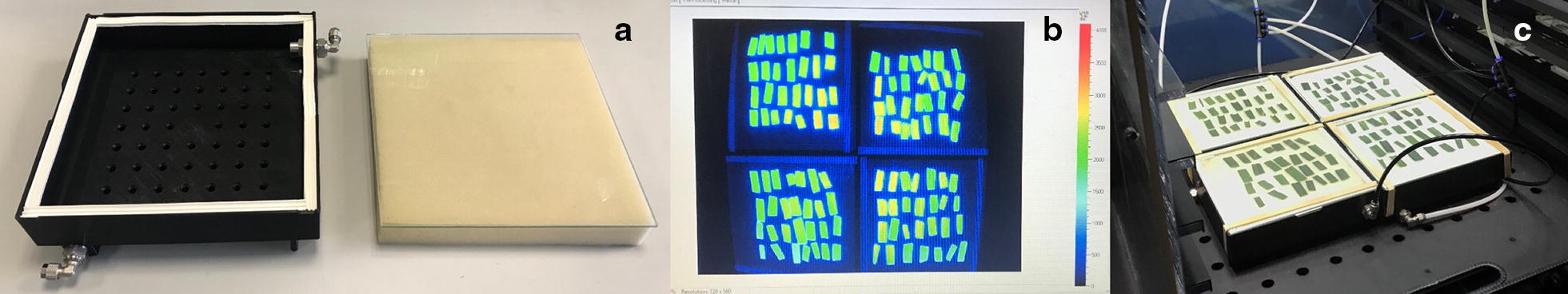
A single Perspex imaging chamber (**a**) with foam insert and glass lid, gas inlet located below perforated divider plate and sampling outlet located at sample height to monitor gaseous conditions within the chamber. This single chamber can be imaged alone or (**b**) in combination with three additional chambers to fill the space below the FluorCam camera. The chambers shown in (**b**) are in-house designed and 3D printed in plastic resin (see Imaging Chamber Design). In this example (**c**), ~ 120 flag leaf sections were imaged at once in response to a custom protocol. With 1 h dark adaption and a 1 h protocol, it is possible to image up to 480 samples if measuring 8 am–3 pm

In order to control concentrations of O_2_ and CO_2_ around the leaf sections, four chambers were designed and constructed in-house (Figs. 2 and 3). The chambers measure 20 cm (length) × 20 cm (width) × 10 cm (depth). A separate, perforated divider halves the chamber (Fig. 3a), forcing gas flow over an even area and through a high-density sponge soaked in water (2.8 mm thick). This sponge sits on the divider and humidifies the dry, cylinder-supplied air. A rubber seal on the top edge of the chamber provides a better gas seal to stabilise gaseous conditions in the chamber. Leaf sections are positioned, adaxial side-up, on the dampened sponge and a glass plate laid on top to produce a flat surface to image. Rubber bands provide further compression around each side of the chamber and further stabilise the concentration of gases at leaf level. Gas enters through 6 × 4 mm (OD × ID) PTFE tubing via a 6 mm (OD) pneumatic fitting located in the lower half of the chamber. To monitor gas concentrations at leaf level, a second 6 mm fitting is located in the top half the chamber at the furthest point from gas entry. The chambers allow imaging of approximately 25–30 mature *T. aestivum* flag leaf sections (2 cm × 3 cm) per chamber—in total 100–120 samples per measurement session is all four chambers (Fig. 3b) are used (Fig. 3c). Separate gas cylinders (British Oxygen Company, UK) supply either synthetic pressurised air (approximately containing 400 µmol mol^-1^ CO_2_ and 210 mmol mol^−1^ O_2_) or a pre mixed 20 mmol mol^−1^ oxygen, 400 µmol mol^−1^ CO_2_ in nitrogen. As an alternative to the synthetic or pre-mixed gases, mass flow controllers with cheaper industrial standard gases could be used instead. Gas flow was manipulated manually to maintain stable gaseous conditions within the chambers. Chamber CO_2_, O_2_ and pressure were monitored using the calibrated sensors (S102 and S151, Qubit Systems, Kingston, Canada). Flow to the sensors was determined using a rotameter to maintain flows at 500 ml min^−1^. Data was logged using Logger Pro. (Vernier, Coalville, UK) and the sampling time adjusted depending on experiment; typically measurements were taken every 15 s throughout a protocol.

The chambers were designed using Fusion 360 (Autodesk, San Rafael, CA, USA) and 3D printed using an Ultimaker S5 printer in Ultimaker Tough PLA (Ultimaker, Geldermalsen, Neatherlands). Four separate chambers were printed and are designed to be used as separate chambers (i.e. filling with different gas concentrations simultaneously) or as a single unit (i.e. all chambers filling with the same gas concentration). This flexibility allows application of a highly controllable screen for the response of photosynthetic traits to complex environmental fluctuations at same time but also provides a high throughput screen when phenotyping large populations of plants.

The CAD files for the final chamber design are fully available with this manuscript (Additional file 2) including printer settings and additional notes, so that users can either print their own, outsource the printing or modify the designs. The design could also be constructed using Perspex sheets if access to a 3D printer is not available (written instructions and necessary tools not listed).

### Plant material and cultivation

The modern wheat cultivars *T. aestivum* ‘Paragon’, ‘Pavon76’ and ‘Highbury’, the landrace *T. aestivum* ‘CS94’ were grown (April–October 2017) in a glasshouse at Sutton Bonington, Leicestershire, UK. Glasshouse conditions were maintained at 22–18 °C ± 2 °C (day/night) under regular mildew, aphid, and thrips control measures applied following the manufacturers’ recommendations. Plants received supplemental lighting (Son-T, Philips, Surrey, UK) to 16 h light or when PPFD fell below 500 µmol m^−2^ s^−1^. For comparing live leaf samples to excised leaf samples, 3 week old seedlings grown in compost (Levington F2S, Scotts, Bramford, UK) filled 12 well trays. To analyse larger leaf sections plants were grown individually in 2L pots, automatically irrigated twice daily for approximately 1 min with feed (HortiMix Standard, Hortifeeds, Lincoln, UK).

### Description of measurements

The white LED light was used as the actinic source for all protocols described in this paper (Additional file 1: Fig. S1). All samples were dark adapted inside the chamber for 1 h before a saturating pulse (5500 µmol m^−2^ s^−1^ PPFD, duration 800 ms) was taken to measure *F*_v_/*F*_m_. Any increases in PPFD occurred immediately after this measurement.

In order to assess any physiological differences between the responses of whole leaves and excised leaf sections, 3 week old seedlings of *T. aestivum* ‘Paragon’ and leaf sections of the same age were measured simultaneously using the FluorCam and a single imaging chamber. Leaf sections were excised in the glasshouse, immediately placed between dampened tissues and placed in an insulated box for transportation to the lab. In the lab, the sections were aligned on damp sponge while live material was carefully tucked under the glass top plate of the chamber. To determine the length of time that leaf samples could be measured after excision without a negative impact on PSII, light response curves were conducted every hour for 9am to 5 pm allowing a 1 h dark adaption period between measurements. The light curve protocol consists of six subsequent saturating pulses taken every 60 s at the end of a step-wise increases from 26 to 1140 µmol m^−2^ s^−1^ PPFD. Prior to every light response curve, a measurement of *F*_v_/*F*_m_ was taken.

To determine variation between modern wheat bread wheat cultivars, *T. aestivum* cv.’Paragon’, ‘Pavon76’ and the landrace *T. aestivum* ‘Chinese Spring 94’ were grown. Flag leaf sections (GS40-41) were excised at 9 am and allowed to dark-adapt for 1 h in the imaging chamber. Fifteen minutes before the initial saturating pulse to determine *F*_v_/*F*_m_ was taken, ambient air was used to saturate the chamber to ensure consistent CO_2_ and O_2_ concentrations around the samples. The protocol consisted of three consecutive light-steps of 15 min; 500 µmol m^−2^ s^−1^, 100 µmol m^−2^ s^−1^ and 1000 µmol m^−2^ s^−1^ PPFD. Saturating pulses were taken every minute throughout the protocol.

**Fig. 4:**
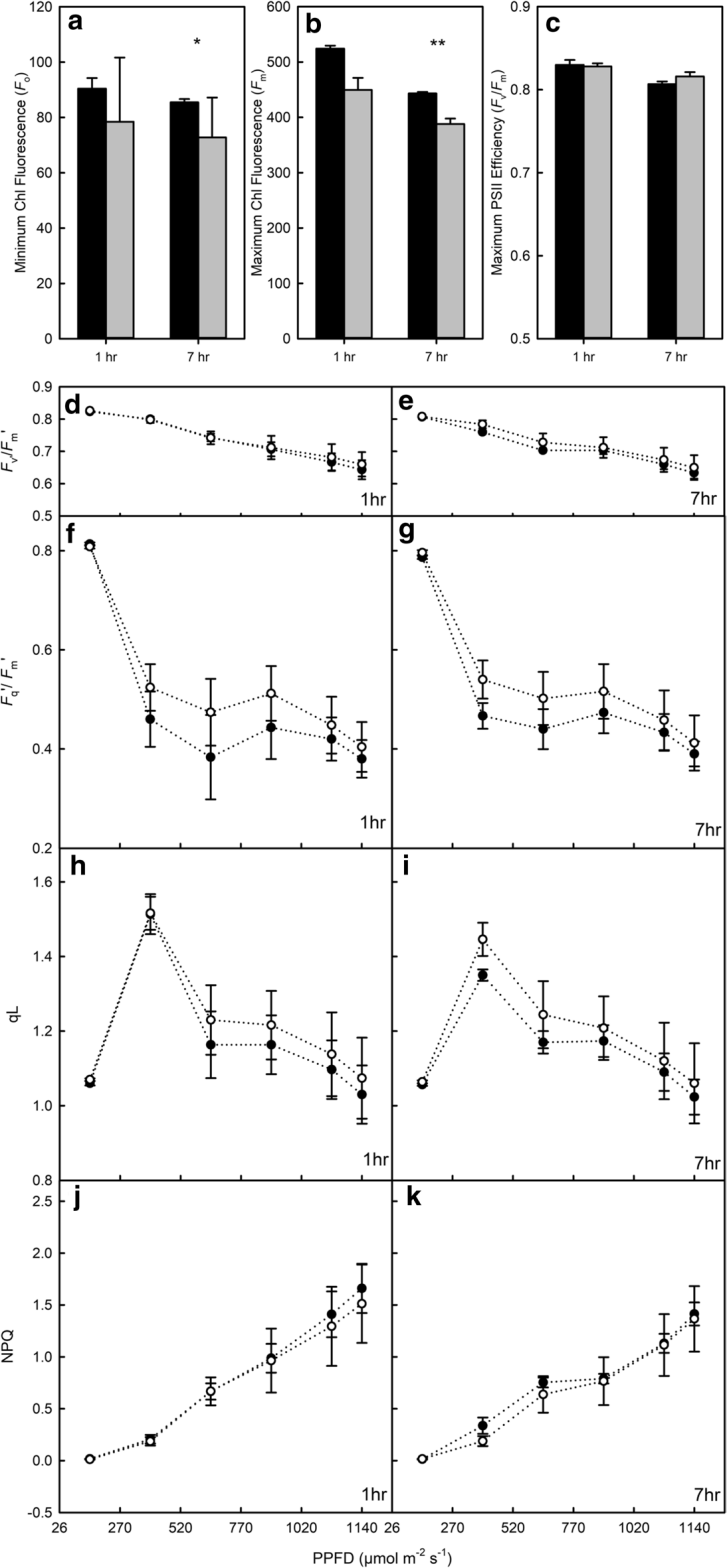
Minimum (**a**—*F*_o_) and maximum (**b**—*F*_m_) fluorescence signal after 1 and 7 h in the imaging chamber for intact leaf material (black) and excised (grey) leaf tissue. From these values, the maximum PSII efficiency (**c**—*F*_v_/*F*_m_) was calculated. Subsequent measurements of maximum PSII efficiency in the light (*F*_v_′/*F*_m_′, **d** and **e**), PSII operating efficiency (*F*_q_′/*F*_m_′—**f** and **g**), the fraction of open PSII centres (qL—**h** and **i**) and non-photochemical quenching (NPQ—**j** and **k**) were calculated in response to increasing light intensity for excised (white circle) and intact (black circle) leaf material (n = 3–5) for 1 and 7 h after sampling. See Table 1 for full descriptions of chlorophyll fluorescence parameters. Asterisks indicate significant differences between excised and intact tissue, where ‘***’*P *< 0.001 ‘**’*P *< 0.01 ‘*’*P *< 0.05

To determine the stability and ease of switching between different gas concentrations in the chambers, four chambers were connected to a compressed synthetic air gas supply (British Oxygen Company, UK), and sealed using the glass lids and bands (Figs. 3 and 4). The gases were turned on and O_2_, CO_2_ and atmospheric pressure were monitored every 15 s. After 10 min, the synthetic gas supply was switched to a commercial pre-mix 2% O_2_ (20 mmol mol^−1^ O_2_, 385 µmol mol^−1^ CO_2_ and N_2_ balance—British Oxygen Company, UK). After three 10 min switches, a 15 min period of each of the gases was demonstrated.

Finally, to ascertain the impact of low oxygen on chlorophyll fluorescence parameters, flag leaf sections were harvested from *T. aestivum* ‘Pavon76’ at 9am and exposed to either compressed synthetic air (180 mmol mol^−1^ O_2_ and 380 µmol mol^-1^ CO_2_, N_2_ balance) or 2% oxygen (20 mmol mol^−1^ O_2_, 385 µmol mol^−1^ CO_2_ and N_2_ balance). Forty-five minutes into the dark adaption the gases were turned on, flooding the chamber. Gaseous conditions inside the chamber were allowed to stabilise for 15 min. *F*_v_/*F*_m_ was measured, followed by a light response curve. The values of *F*_v_/*F*_m_ and the responses of *F*_v_′/*F*_m_′, *F*_q_′/*F*_m_′, qP and NPQ were extracted from each protocol.

### Determining the rates of relaxation and induction in NPQ

In order to determine the rate of NPQ relaxation (Eq. 1) and induction (Eq. 2) data was fitted using a 2-factor exponential decline to minimum (Eq. 1) or rise to maximum (Eq. 2) using curve fitting toolbox in Matlab (Matlab R2018a, USA).

1 $$y = a \cdot e\left( { - b \cdot x} \right)$$

2 $$y = a \cdot e^{ - bx}$$

where *a* is the initial value and *b* a constant representing the rate of exponential decay or growth. In order to determine the time (*t*) taken to achieve either 50% of the maximum NPQ values (I_50_) or 50% of the minimum NPQ values (R_50_) the equations were solved for *b* and the following calculations applied;

$$t = 1/b$$

$$t*\ln \left( 2 \right)$$

where *t* is the time constant and *b* is the rate obtained from the rearrangement of either Eq. 1 or Eq. 2.

### Statistical analyses

Statistical analyses were conducted in R (http://www.r-project.org/). A Shapiro–Wilk test was used to test for normality and a Levene’s test of homogeneity was used to determine if samples had equal variance. Single factor differences were analysed using a one-way ANOVA with a Tukey–Kramer honest significant difference (HSD) test where more than one group existed or using a Student’s *t* test where only two groups were compared.

## Results

### Comparing the diurnal response of intact leaf material with leaf sections

In order to assess physiological differences between the responses of whole leaves and excised leaf sections, seedlings of *T. aestivum* ‘Paragon’ and leaf sections of the same age were measured simultaneously using the FluorCam (Fig. 4) over a 7 h time period. After the first dark adaption period, the intact material demonstrated no significant difference in *F*_o_ and *F*_m_ respectively compared to the excised material (Fig. 4a, b). However, it is important to note that the trend in these constituents of F_v_/F_m_ was consistent throughout the day regardless of treatment (Fig. 4c). After 7 h in the chamber, values of *F*_m_ and *F*_o_ were significantly (*P* < 0.05) higher in the intact leaf tissue, however this did not lead to a significant difference in *F*_v_. Similarly, when comparing *F*_v_/*F*_m_ values 1 h and 7 h after sampling, there was no difference in values between the intact or excised leaves (*P* = 0.79). Immediately after measuring *F*_v_/*F*_m_, a light response curve was taken. During the curve, a general decrease in maximum PSII efficiency in the light (*F*_v_′/*F*_m_′—Fig. 4d, e), PSII operating efficiency (*F*_q_′/*F*_m_′—Fig. 4f, g) and the fraction of open PSII centres (qL—Fig. 4h, i) was observed for the leaf treatments. Non-photochemical quenching (NPQ—Fig. 4j, k) increased with increasing PPFD But with no significant differences observed over time for any of the response parameters measured during the light response curve (*P* > 0.2). No significant differences were determined between the responses measured 1 h or 7 h after sampling when comparing just the intact leaves or the excised leaf tissue (*P* > 0.081).

### Identifying variation in the response of PSII for cultivars of *T. aestivum*

**Fig. 5:**
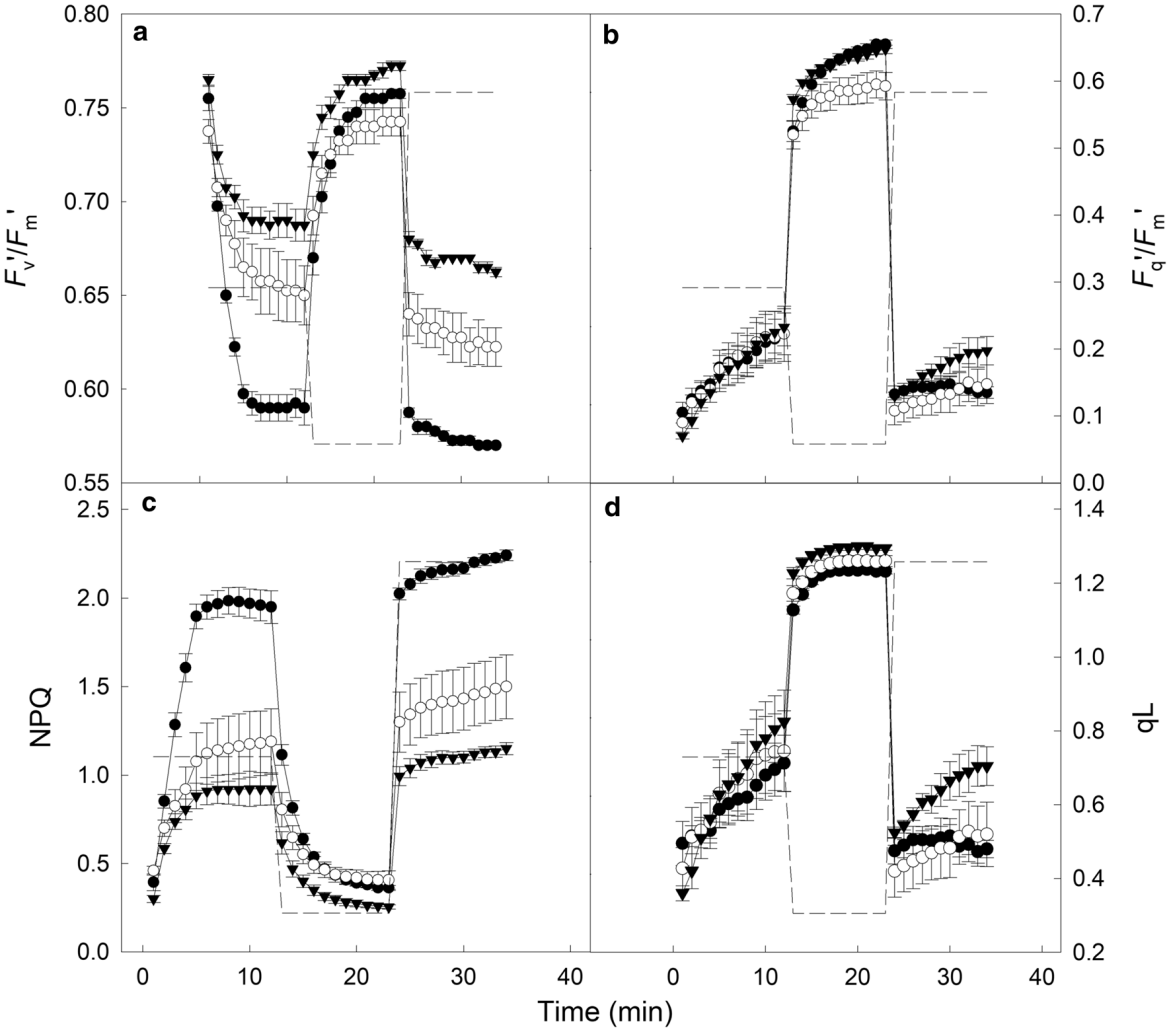
The response of chlorophyll fluorescence (see Table 1 for full descriptions) to stepwise changes in photosynthetic photon flux density (--- PPFD) in two modern bread wheat cultivars—*T. aestivum* ‘Paragon W07/08’ (black circle) and *T. aestivum* ‘Pavon76’ (inverted black triangle)—and landrace *T. aestivum* ‘CS94’ (white circle). After a dark adaptation period of 1 h, PPFD was increased to 500 µmol m^−2^ s^−1^ for 15 min. Subsequently, PPFD was decreased to 100 µmol m^−2^ s^−1^ for 10 min and then increased to 1000 µmol m^−2^ s^−1^ for 10 min. From measurements of maximal (*F*_m_) and minimal (*F*_o_) fluorescence the following parameters can be calculated: operating efficiency in the light (**a**—*F*_v_’/*F*_m_’), PSII quantum yield of PSII (**b**—*F*_q_’/*F*_m_’), maximum non-photochemical quenching (**c**—NPQ) and fraction of open PSII reaction centres (**d**—qL). Measurements were taken every minute and error bars indicate standard error (n = 4)

To identify variation in the magnitude of change and kinetic response of PSII, leaf sections from *T. aestivum* ‘Paragon W07/08’, ‘Pavon76’ and ‘CS94’ were subjected to step changes in PPFD after an initial measurement of *F*_v_/*F*_m_ (Fig. 5). No significant differences (*F*_(2,9)_ = 0.36, *P* = 0.705) were observed between the cultivars *F*_v_/*F*_m_, with all leaf sections achieving 0.81 ± 0.01. When illuminated with 500 and 1000 µmol m^−2^ s^−1^ PPFD, ‘Pavon76’ achieved significantly higher (*P* < 0.03) values of *F*_m_ when compared to the other modern cultivar ‘Paragon W07/08’ (data not shown). Following the measurement of *F*_v_/*F*_m_, the leaf sections were exposed to 500 (15 min), 100 (10 min) and 1000 (10 min) µmol m^−2^ s^−1^ PPFD. Maximum PSII efficiency in the light (*F*_v_′/*F*_m_′, Fig. 5a), PSII operating efficiency (*F*_q_′/*F*_m_′—Fig. 5b) and non-photochemical quenching (NPQ—Fig. 5c) reached steady-state in 5–10 min under 500 µmol m^−2^ s^−1^ PPFD while the fraction of open PSII centres (qL—Fig. 5d) never achieved steady state. In general, the response to subsequent light-steps of 100 and 1000 µmol m^−2^ s^−1^ PPFD was more rapid.

During the light-step treatment, ‘Paragon W07/08’ demonstrated the lowest *F*_v_′/*F*_m_′ values under both 500 and 1000 µmol m^−2^ s^−1^ PPFD (Fig. 5a), significantly lower than those measured in ‘Pavon76’ and ‘CS94’ (*F*_(2,9)_ = 17.86, *P* < 0.013). Conversely, ‘Paragon W07/08’ demonstrated the highest values of NPQ (Fig. 5c), significantly higher than those measured for ‘Pavon76’ and ‘CS94’ under 500 and 1000 µmol m^−2^ s^−1^ PPFD (*F*_(2,9)_ = 16.68, *P *< 0.001 and *F*_(2,9)_ = 26.9, *P *< 0.0002 respectively). No significant differences were observed between plants under the two high light steps for *F*_q_′/*F*_m_′ (Fig. 5b), however ‘CS94’ achieved significantly higher values (*P *< 0.04) under 100 µmol m^−2^ s^−1^ PPFD when compared to the modern cultivars ‘Paragon W07/08’ and ‘Pavon76’.

In order to determine differences in the rate of NPQ relaxation (R_50_—500 to 100 µmol m^−2^ s^−1^ PPFD) and induction (I_50_—100 to 1000 µmol m^−2^ s^−1^ PPFD), single factor exponential functions were fitted to sections of the response data (see “Statistical analyses”). In general, all three cultivars showed a slower relaxation of NPQ on transfer to low light compared to the time taken to fully induce NPQ under high light (Table 2) which is generally consistent with the known kinetics of NPQ and its regulation by PsbS and the xanthophyll cycle [33]. Although no significant differences were noted for either I_50_ or R_50_ between cultivars, a general trend was observed that cultivars with higher NPQ values under high PPFD took longer to relax when light intensity was decreased. Modern cultivar ‘Paragon W07/08’ showed the highest values in NPQ under 1000 µmol m^−2^ s^−1^ PPFD (*P* < 0.04), took the longest to relax under 100 µmol m^−2^ s^−1^ PPFD and demonstrated the greatest percentage decline in magnitude (Table 2). Conversely, the lowest values of NPQ under 100 and 1000 µmol m^−2^ s^−1^ PPFD were observed for ‘Pavon76’, which relaxed in the shortest amount of time but took over 3 s longer to achieve half the maximum NPQ under 1000 µmol m^−2^ s^−1^ PPFD when compared to ‘Paragon W07/08’ or ‘CS94’.

**Table 2:** Response of non-photochemical quenching to step-decreases (relaxation) and increases (induction) in PPFD

Cultivar	Relaxation *500 to 100 umol m* ^− *2*^ *s* ^− *1*^ *PPFD*			Induction *100 to 1000 umol m* ^− *2*^ *s* ^− *1*^ *PPFD*		
	Min. NPQ	R_50_ (s)	Decrease in NPQ (%)	Max. NPQ	I_50_ (s)	Fold increase
Paragon W07/08	0.37 ± 0.018	124.5 ± 6.6	81.3 ± 0.7*	2.2 ± 0.0*	5 ± 1.4	6.2 ± 0.3*
CS94	0.43 ± 0.058	108.6 ± 5.8	64.8 ± 3.2	1.5 ± 0.3	4.1 ± 1.3	3.7 ± 0.5
Pavon76	0.26 ± 0.012	67.9 ± 1.0	71.9 ± 1.8	1.2 ± 0.0	9 ± 1.5	4.5 ± 0.2

Example parameters extracted from the response of non-photochemical quenching (NPQ) in leaf sections of *T. aestivum* ‘Paragon W07/08’, ‘CS94’ and ‘Pavon76’ shown in Fig. 5. Measurements were taken every minute under step-wise changes in Photosynthetic Photon Flux Density (PPFD) from 500 (15 min), 100 (10 min) to 1000 umol m^−2^ s^−1^ (10 min). The minimum and maximum NPQ values were determined under 100 and 1000 umol m^−2^ s^−1^ PPFD respectively. In addition, the percentage decrease (500 to 100 umol m^−2^ s^−1^ PPFD) and fold-increase (100–1000 umol m^−2^ s^−1^) in NPQ were calculated. Lastly, a 2-factor exponential decay function was used to determine the time taken to achieve half the minimum (relaxation—R_50_) or maximum (induction—I_50_) NPQ (see’*Determining the rates of relaxation and induction in NPQ′*). Data are the mean ± SE (n = 3–4). Asterisks indicate significant differences where ‘***’ *P *< 0.001 ‘**’ *P *< 0.01 ‘*’ *P *< 0.05

### Timing and control of gases inside the imaging chamber

At 20 mmol m^−2^ O_2_, photorespiration is inhibited and the fluorescence parameter *F*_q_′/*F*_m_′ is linearly related to the rate of photosynthetic CO_2_ uptake [23, 25]. Therefore, it was essential that that [O_2_] within the chamber was (a) stable and (b) able to be manipulated to rapidly switch between atmospheric [O_2_] to 20 mmol m^−2^. As an example, during the measurement of typical light response curve, the chosen [O_2_] would have to be maintained for approximately 15 min while CF images are being taken.

**Fig. 6:**
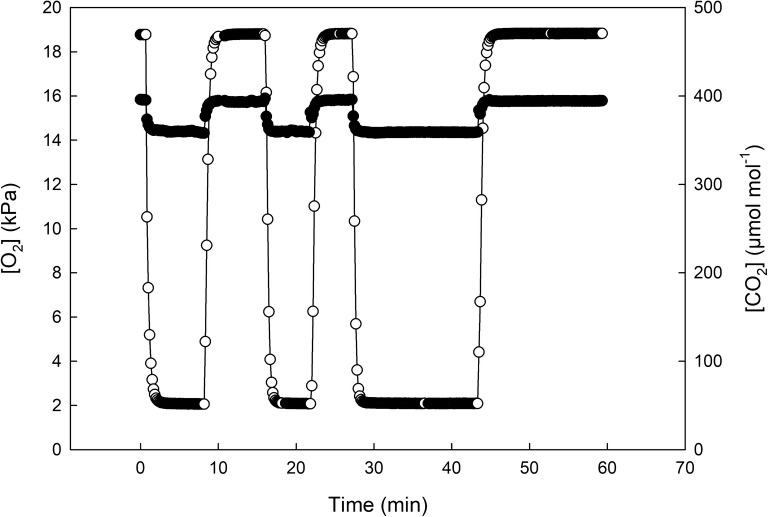
Concentrations of O_2_ (white circle) and CO_2_ (black circle) in the measuring chamber during an experiment. Measurements were taken every 15 s. Oxygen concentration was switched from ambient compressed air (19 kPa) to 2 kPa three times while maintaining 375 ± 18 µmol mol^−1^ CO_2_

In the example shown in Fig. 6, low [O_2_] was achieved in < 120 s and was maintained within 1% of the target value for several minutes before returning to near-ambient conditions within 135 s. After three 10 min switches, a 15 min period of both low [O_2_] and ambient [O_2_] are demonstrated. Figure 6 also illustrates the stability of [CO_2_] (376 µmol mol^−1^ ± 4.5%) within the chamber.

### Screening leaf sections under ambient and low oxygen

**Fig. 7:**
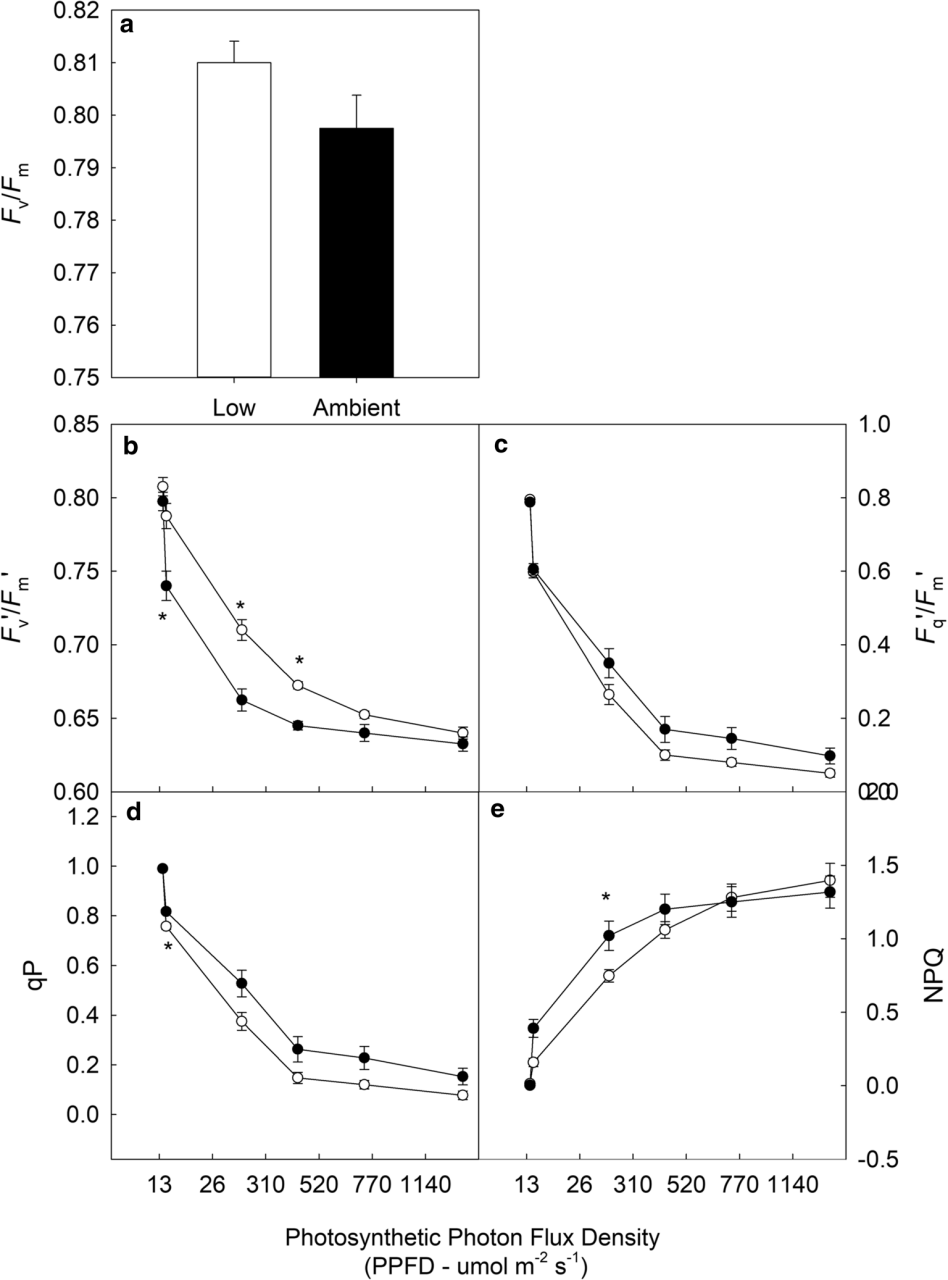
The response of chlorophyll fluorescence parameters (see Table 1 for full descriptions) in the wheat cultivar *T. aestivum* ‘Pavon76’ to stepwise increases in Photosynthetic Photon Flux Density (PPFD) from 0 to 100% (0–1027 umol m^−2^ s^−1^ PPFD) under 20 (white circle) or 200 (black circle) mmol mol^−1^ O_2_. After a dark adaptation period of 1 h, maximum PSII efficiency was determined (**a**—*F*_v_/*F*_m_). From the dark-adapted values of maximal (*F*_m_) and minimal (*F*_o_) fluorescence, the following parameters can be calculated: operating efficiency in the light (**b**—*F*_v_′/*F*_m_′), PSII quantum yield of PSII (**c**—*F*_q_′/*F*_m_′), fraction of open PSII reaction centres (**d**—qP) and non-photochemical quenching (**e**—NPQ) and. Data are the mean ± SE (n = 4). Asterisks indicate significant differences where ‘***’*P *< 0.001 ‘**’*P *< 0.01 ‘*’*P *< 0.05

Under 20 mmol mol^−1^ O_2_, photorespiration is inhibited and CO_2_ assimilation represents the major sink for the end-products of electron transport (ATP and NADPH) [20, 40]. Leaf sections of *T aestivum* cv.’Pavon76’ were imaged under low and ambient O_2_ concentrations (see Fig. 7). The maximum quantum efficiency of PSII photochemistry (*F*_v_/*F*_m_—Fig. 7a), maximum quantum efficiency of PSII photochemistry in the light (*F*_v_′/*F*_m_′—Fig. 7b), *F*_q_′/*F*_m_′ (Fig. 7c), photochemical quenching (qP—Fig. 7d) and NPQ (Fig. 7e) were measured in response to stepwise increases in PPFD from 0 to 1140 umol m^−2^ s^−1^. These responses were first measured under 20 mmol mol^−1^ O_2_ followed by 200 umol mol^−1^ O_2_. No significant difference (P = 0.15) was observed between *F*_v_/*F*_m_ values under low or ambient O_2_ (Fig. 7a). Similarly, during the protocol, no significant differences were noted for *F*_m_′ (*P *> 0.061) or *F*_o_′ (*P *> 0.31). In general, values of *F*_v_′/*F*_m_′ were higher under low oxygen, with measurements taken under 520 μmol m^−2^ s^−1^ being significantly higher (*P *< 0.05) than the values obtained under ambient oxygen concentrations. In general, values of *F*_q_′/*F*_m_′, qP and NPQ were lower under low oxygen conditions but these differences were only significant at low light intensities for NPQ and qP.

## Discussion

This study describes the amalgamation of high throughput PAM-based chlorophyll fluorescence imaging with rapid sampling and screening of wheat (*T. aestivum)* leaf sections under controlled gaseous environments. This methodology provides the means to rapidly phenotype high numbers of plants for changes in the state of PSII under controlled environmental conditions and in response to user-determined PPFD perturbations. The work presented in this paper demonstrates that excised leaf tissue is representative of intact material for such photosynthetic assays (Fig. 4) and has potential to contribute to technologies to alleviate the bottleneck in phenotyping large populations of glasshouse or field grown plants.

### Excised wheat leaf material is representative of intact material

Numerous physiological measurements still employ leaf sections as a means to determining variation between plants [41–43], and while invasive, the imaging of leaf sections increases the number of individuals measured at one developmental stage and at one time point. The user is less limited by the size of the CF imager and more by the number of plants that can be grown at the facility—making this system a cost effective, high throughput option for phenotyping plant populations.

As shown in Fig. 4, the response of excised leaf tissue was representative of the intact leaf material, both in response to PPFD and over the 9 h measurement period. This has important implications in logistics of screening large populations—allowing researchers to sample, transport and measure material in the timeframe of a day without the worry of sample degradation. Maintaining on damp tissue at the temperature of sampling (~ 20–24 °C) seems to be the optimal method of transporting the lamps to the CF imager, while cooling samples (< 10 °C) may have negative implications for photosynthesis [44, 45] and leaf metabolite composition [46] over time. We suggest that a further development of this technique is a simplified design that is suitable for moving to a field location. This would require an appropriate power supply.

### Flexible screening technologies improve selection power

The development of custom, semi-gas tight chambers to arrange the samples increases the capability of the system, providing the means to screen under non-photorespiratory conditions or to supply a custom gas mix e.g. anaerobic conditions or saturating CO_2_. Assessment of photosynthetic processes under different gas mixtures adds extra strength to the screen. For example, at low (20 mmol mol^−1^) oxygen, the relative contribution of photorespiration can be estimated and a linear relationship between *F*_q_′/*F*_m_′ and the quantum yield of CO_2_ assimilation (and therefore linear electron flux) can be accurately monitored between samples [20, 25, 28]. When imaged at low oxygen, the excised leaf sections had higher values of *F*_v_′/*F*_m_′, which were significantly higher at the majority of light intensities, but lower values of *F*_q_′/*F*_m_′ and qP (Fig. 7). This data suggests that under low oxygen, the leaf sections exhibited greater maximum PSII efficiency that was not realised in the other CF parameters for this cultivar. This implies variation in the capacity of PSII to assimilate CO_2_, a trait which can be further investigated using traditional techniques (such as IRGAs) to determine, for example, Rubisco kinetics.

Coupling this technology with other imaging technologies to increase the overall predictive power of the system e.g. screening for absorbance, reflectance indices, and stomatal density. For example, it has been shown that glaucousness can decrease light absorbance in barley leaves by up to 12% [47], therefore limiting light acquisition and decreasing leaf temperature under the lower light levels experienced in the canopy [48]. Accounting for this developmental disparity in a large population of high leaf index species (such as wheat), perhaps using scoring or reflectance indices and coupling with the method of chlorophyll fluorescence screening discussed here, would increase a breeder or researchers selection power. In this example, the rapid, systematic screening of a selection of leaf-level traits has the potential to aid the identification of lines able to maintain or increase yields under fluctuating environmental conditions experienced in the field. The ability to monitor photosynthetic processes under fluctuating PPFD conditions also provides a powerful tool in determining complex, often hierarchical, photosynthetic responses.

The temporal response of photosynthesis to fluctuating light has gathered growing interest in recent years as the technology for producing rapidly adjustable PPFD environments and PSII monitoring is more accessible to researchers [49]. Plants are subject to changes in spectral quality and intensity of PPFD during the growing season; constantly adapting and acclimating to optimise the conversion of CO_2_ to biomass and, ultimately, yield [49–51]. Whether the photosynthetic acclimation to the PPFD environment is a dynamic (reversible) or developmental (morphological), diversity in light acclimation exists between different species [52] and the methodology described here can provide rapid identification of dynamic differences between large populations of mono-cropped plants, determining individuals which will optimally respond to the light environments and improve identification of potentially high yielding varieties.

For example, the response of NPQ has been previously identified as a target for improvement [53], with the rate of relaxation thought to inhibit CO_2_ fixation between 5.5 and 30% in tobacco [33]. These observations indicated that there is an optimum value in the magnitude and rate of NPQ induction and relaxation for crop species. While rapid up-regulation of NPQ (e.g. over expression of PSII protein, PsbS [41]) may serve to protect a plant during high light or rapidly fluctuating conditions, a slow relaxation or downregulation may inhibit CO_2_ assimilation. The timing of induction and relaxation presented in this paper demonstrated a significant degree of variability in the time taken to induce and relax NPQ (Table 2) however, it was noted a line which achieved faster inductions under high light did not necessarily achieve a faster relaxation under low light. The term NPQ encompasses mechanisms employed by the photosynthetic machinery to regulate the thermal dissipation of excess excitation energy [20]. These processes are mainly associated with activity of the xanthophyll cycle [54, 55], PsbS and protonation of PSII antennae proteins [56]. The data presented in this paper suggests there is variation to be exploited for improvement, especially as pre-breeding and breeding programs are now generating cultivars with wild relative or land race genomic introgressions as a means to improve genetic diversity in our modern varieties [57, 58]. Phenotyping using the screening method discussed in this paper has a two-fold role; the identification of variation within the generated population and linking that variation to a wild or landrace parent.

Extrapolating both steady-state and the timing of chlorophyll fluorescence parameter responses (e.g. NPQ) highlight a rapid and simple method of screening high numbers of plants for differences in the magnitude and rate of response of PSII to PPFD. From this data, efforts can be concentrated on larger populations of smaller numbers of lines for the greatest differences in photosynthesis. Here we provide one example: the Watkins core collection is a set of genetically diverse landraces consisting of 119 accessions. Using the method outlined in this paper it would take between 1 and 2 days to perform a simple PPFD-based screen on a population of 476 plants (assuming four replicates). To increase the efficiency of this process a seedling screen could be performed prior to vernalisation (therefore cutting down on valuable glasshouse and growth room space), and then again at physiologically relevant growth stages; flag leaf, booting, anthesis and post anthesis. There is no other technique to our knowledge that is capable of such throughput and resolution in terms of dynamic photosynthesis and photoprotection.

## Conclusions

The methodology described here demonstrates a rapid, novel high throughput approach for phenotyping photosynthetic processes in a major crop species under controlled PPFD and gaseous conditions. We have demonstrated that excised tissues can be used as a robust proxy for intact leaf material and, with this method, exposed cultivar-specific responses to dynamic PPFD protocols. Furthermore, we have built and tested custom imaging chambers, capable of maintaining gaseous concentrations around excised leaves during protocols to provide the user control over this additional environmental variable. This overcomes logistical and practical problems of rapidly applying such sophisticated dynamic light protocols during CF analyses on large plants such as cereals. Finally, we have discussed the logistics of screening > 500 samples per day using this methodology to contribute a wealth of phenotypic data and improve selection power in large populations of wheat with the ultimate aim of improving yield through improved photosynthesis and RUE.

## Supplementary information

**Additional file 1: Figure S1.** The spectrum of the white (-) and measuring (…) light sources used with the FluorCam. The ‘white’ light source peaks at 448 and 553 nm while the measuring light peaks at 617 nm. Spectra shown were measured as the average of 10 spectrums.

**Additional file 2.** Supplementary information and files on the design and construction of the custom imaging chambers.

## Data Availability

The datasets analysed during the current study are available from the corresponding author on reasonable request.
