# Supplementary figures and images for: High throughput procedure utilising chlorophyll fluorescence imaging to phenotype dynamic photosynthesis and photoprotection in leaves under controlled gaseous conditions
Source: Plant Methods. 2019 Sep 18;15:109. doi: 10.1186/s13007-019-0485-x (PMC6749646; doi:10.1186/s13007-019-0485-x)

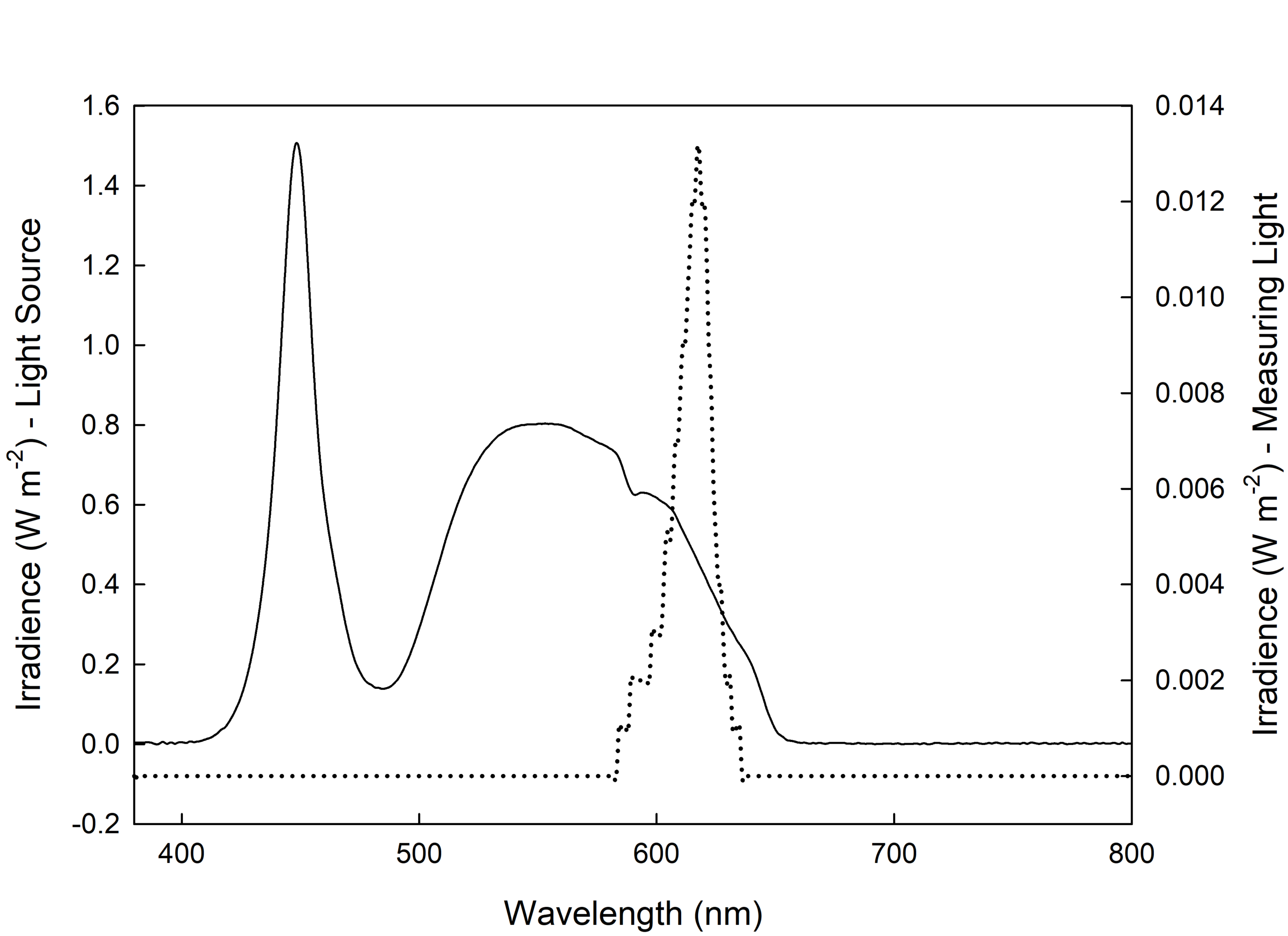

Supplement: Supplementary file 1 — Additional file 1: Figure S1. The spectrum of the white (-) and measuring (…) light sources used with the FluorCam. The ‘white’ light source peaks at 448 and 553 nm while the measuring light peaks at 617 nm. Spectra shown were measured as the average of 10 spectrums. [file 13007_2019_485_MOESM1_ESM.png]
