# Supplementary material for: High throughput procedure utilising chlorophyll fluorescence imaging to phenotype dynamic photosynthesis and photoprotection in leaves under controlled gaseous conditions
Source: Plant Methods. 2019 Sep 18;15:109. doi: 10.1186/s13007-019-0485-x (PMC6749646; doi:10.1186/s13007-019-0485-x)
Supplement: Supplementary file 2 — Additional file 2. Supplementary information and files on the design and construction of the custom imaging chambers. [file 13007_2019_485_MOESM2_ESM.zip › Additional_file_2/design_labelled.pptx]

## Slide 1
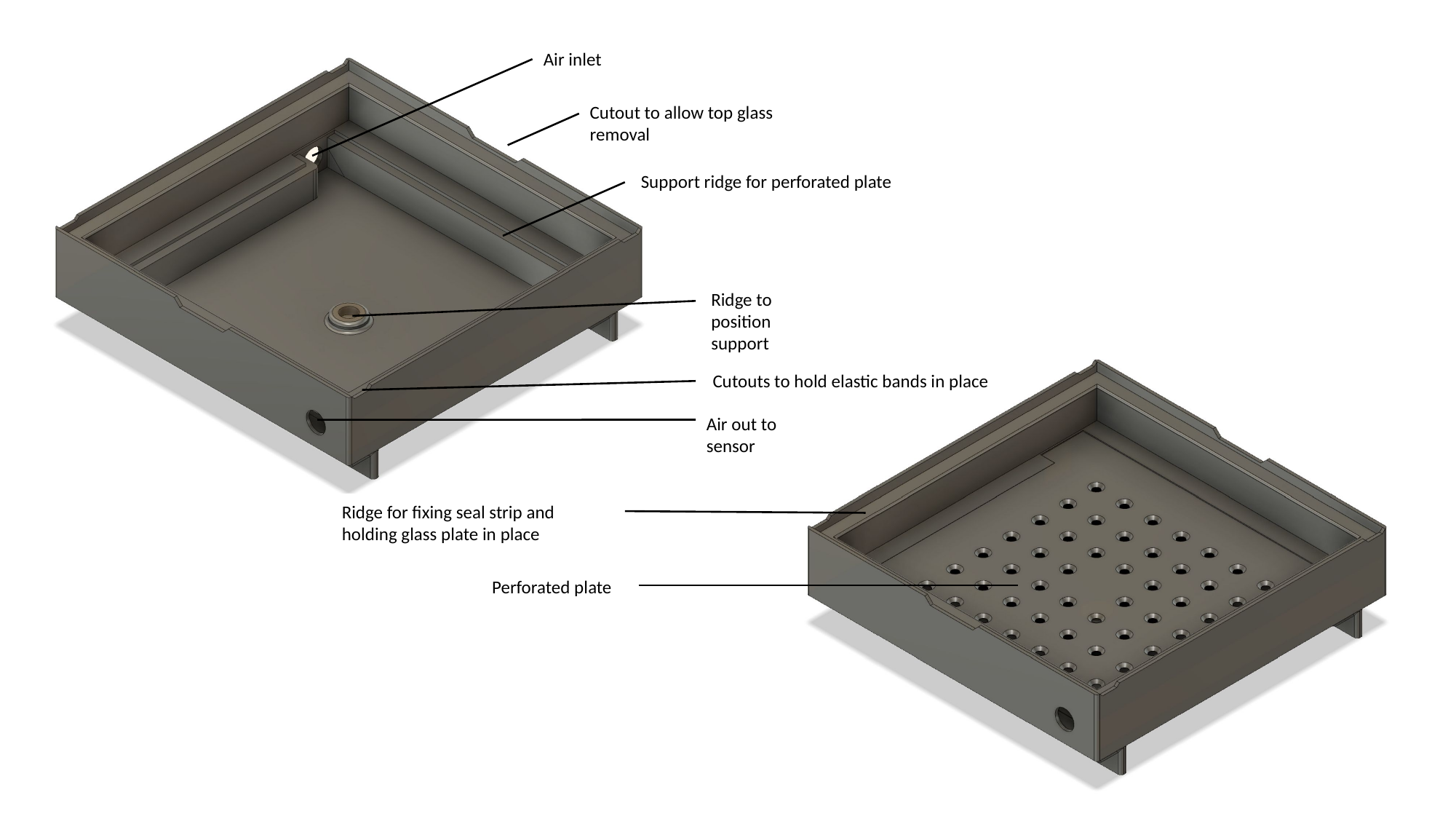

Air inlet
Cutout to allow top glass removal
Support ridge for perforated plate
Ridge to position support
Cutouts to hold elastic bands in place
Air out to sensor
Ridge for fixing seal strip and holding glass plate in place
Perforated plate
