# Supplementary material for: High throughput procedure utilising chlorophyll fluorescence imaging to phenotype dynamic photosynthesis and photoprotection in leaves under controlled gaseous conditions
Source: Plant Methods. 2019 Sep 18;15:109. doi: 10.1186/s13007-019-0485-x (PMC6749646; doi:10.1186/s13007-019-0485-x)
Supplement: Supplementary file 2 — Additional file 2. Supplementary information and files on the design and construction of the custom imaging chambers. [file 13007_2019_485_MOESM2_ESM.zip › Additional_file_2/read_me.rtf]

Imaging Chamber The chambers are printed in 3 parts (the main chamber body, a perforated middle layer and a support stub to prevent the middle layer from flexing. The purpose of this design was to allow access into the bottom chamber for cleaning. All 3 parts can be printed separately or together depending on the size and shape of the 3D printer. Indicative settings below are for an Ultimaker S5 using Ultimaker Tough PLA (polylactic acid) as the material and Cura software for slicing. Other printers and materials would work equally well. Layer Height – 0.2mmWall thickness - 1.2mmWall line count – 3Top/Bottom thickness – 1mmInfill Pattern – ‘Lines’Infill Density – 20%Print Speed – 45mm/s The total material weight used is 323g per chamber using these settings. At the time of writing (March 2019), it would cost approximately £78 for the materials required to print four chambers in Tough PLA. File list: Chamber.stl – Main chamberPlate.stl – Middle perforated plateSupport.stl – Central supportChamber.f3d – Main chamber.Plate.f3d – Middle perforated plateSupport.f3d – Central support 
